# Hospitalization for Invasive Pneumococcal Diseases in Young Children before Use of 13-Valent Pneumococcal Conjugate Vaccine, Suzhou, China

**DOI:** 10.3201/eid2701.181415

**Published:** 2021-01

**Authors:** Kaile Chen, Xiyan Zhang, Yunzhen Tao, Yunzhong Wang, Jian Xue, Changpeng Liu, Shuang Feng, Yongdong Yan, Qinghui Chen, Jianmei Tian, Genming Zhao, Xuejun Shao, Tao Zhang

**Affiliations:** Fudan University, Shanghai, China (K. Chen, X. Zhang, C. Liu, S. Feng, G. Zhao, T. Zhang);; Key Laboratory of Public Health Safety, Ministry of Education, Shanghai (K. Chen, X. Zhang, C. Liu, S. Feng, G. Zhao, T. Zhang);; Soochow University Affiliated Children’s Hospital, Suzhou, China (Y. Tao, Y. Wang, J. Xue, Y. Yan, Q. Chen, J. Tian, X. Shao)

**Keywords:** Invasive pneumococcal diseases, Streptococcus pneumoniae, incidence of hospitalization, Suzhou, China, hospitalization, bacteria, streptococci, meningitis, vaccines, pneumococcal conjugate vaccines, PCV7, PCV13

## Abstract

A 13-valent pneumococcal conjugate vaccine against invasive pneumococcal disease (IPD) was introduced in China in April 2017. We describe 105 children <5 years of age who were hospitalized for IPD at Soochow University Affiliated Children’s Hospital in Suzhou, China, during January 2010–December 2017. We calculated the incidence of hospitalization for IPD as 14.55/100,000 children in Suzhou. We identified 8 different capsular serotypes: 6B (28.4% of cases), 14 (18.9% of cases), 19A (18.9% of cases), 19F (12.2% of cases), 23F (10.8% of cases), 20 (4.1% of cases), 9V (4.1% of cases), and 15B/C (2.7% of cases). These results provide baseline data of IPD before the introduction of this vaccine in China, enabling researchers to better understand its effects on IPD incidence.

*Streptococcus pneumoniae* infections are a major cause of illness and death in infants and children worldwide, especially in developing countries (*1*). The World Health Organization estimates that pneumococcal diseases cause ≈1.6 million deaths every year, of which ≈1.0 million occur in children <5 years of age (*2*). Invasive pneumococcal diseases (IPDs) such as bacteremic pneumonia, febrile bacteremia, and meningitis often are fatal, and ≈25%–50% of survivors have serious neurological sequelae (*3*). IPD incidence varies among countries and populations (*4*,*5*). Very young or old age, concurrent conditions, malnutrition, poor healthcare, and low socioeconomic status are risk factors for IPD.

The growing resistance of *S. pneumoniae* to common antimicrobial drugs highlights the importance of vaccines in preventing pneumococcal disease (*6*,*7*). In China, vaccines fall under 2 categories: category I vaccines, which guard against diseases such as hepatitis B, polio, and measles, are mandatory, and are provided by the government; and category II vaccines, which are optional and commercially available. The 7-valent pneumococcal conjugate vaccine (PCV7) was licensed as a categoryvaccine in mainland China in 2008; the estimated uptake rate was 2%–7% (*8*,*9*). The 13-valent pneumococcal conjugate vaccine (PCV13) was introduced in mainland China in November 2016 as a category II vaccine. To evaluate the effect of PCV13 on IPD incidence, we describe baseline epidemiologic characteristics of illness, hospitalization, and death associated with the disease. Li et al. evaluated surveillance data from 4 prefecture cities (Jinan, Yichang, Shijiazhuang, and Guigang) and reported that *S. pneumoniae* was the most common cause of bacterial meningitis in children <5 years of age during 2006–2009 (*10*). However, few data exist on the incidence of pneumococcal pneumonia, sepsis, and other manifestations of IPD in different regions of mainland China.

We describe IPD among patients <5 years of age who were treated at Soochow University Affiliated Children Hospital (SCH) in Suzhou, China, during 2010–2017. We used a rapid method of the World Health Organization to estimate the baseline incidence of IPD hospitalization (*11*) among this age group.

## Methods

### Study Site and Catchment Area

The study was conducted in Suzhou, a major city with a population of ≈12 million persons in the southeast area of Jiangsu Province in eastern China. Suzhou consists of 5 municipal districts (Gusu, New and High-Tech, Wuzhong, Xiangcheng, and Industrial Park) and 5 county-level cities. SCH, which is in Gusu, is the only tertiary children’s hospital in Suzhou. In 2016, the hospital recorded ≈1.9 million outpatient and emergency visits and 45,000 hospitalizations. Hospital records indicate that, during 2011–2014, a total of 84.9% of patients <5 years of age with influenza-like illness and 63.3% of patients <5 years of age with meningitis or encephalitis resided in the 5 municipal districts of Suzhou (*12*). According to a 2011 healthcare utilization survey (*13*,*14*), SCH patients accounted for 67.7% of total discharges of children <5 years of age from all 96 hospitals in downtown Suzhou. We defined the catchment area of SCH as the 5 municipal districts of Suzhou; we assumed that 67.7% of children <5 years of age who resided in the catchment areas would be treated at SCH. The study was conducted in accordance with internationally recognized standards for ethical research and was approved by the institutional review boards of the School of Public Health, Fudan University (approval no. 2015-04-0545) and SCH.

### Case Definition, Identification, and Serotyping

We defined IPD patients as those from whom *S. pneumoniae* was isolated from a normally sterile body site such as cerebrospinal fluid (CSF), blood, or pleural fluid. Within 24 hours after admission, the hospital collects CSF specimens from patients with suspected meningitis, defined as acute onset of fever with change in mental status, meningeal signs (e.g., neck stiffness, headache), or both; blood cultures from patients with a temperature of >39.0°C; and pleural fluid from patients with suspected effusion detected in chest radiography or computed tomography scan. The specimens were sent to the laboratory at SCH for bacterial culture <2 hours after collection according to the standard methods of collection, transport, and culturing (*7*). Laboratory physicians identified isolates as *S. pneumoniae* by morphologic features, α-hemolysis, Gram staining, and bile solubility or optochin susceptibility using Oxoid Optochin Discs (Thermo Fisher Scientific, https://www.thermofisher.com).

We collected the *S. pneumoniae*–positive isolates from SCH and sent them to the Key Laboratory of Public Health Safety at Fudan University, Shanghai, for serotyping. We identified the serotypes of pneumococcal isolates using the Quellung reaction with antisera (Statens Serum Institute, https://en.ssi.dk), a multiplex PCR described previously (*15*), or both.

### Demographic Information

We obtained information on the annual population of children <5 years of age in the catchment area during 2010–2017 from the immunization program database at the Suzhou Center for Disease Control and Prevention, which serves all residents and >95% of the migrant population in this age group in Suzhou. Almost all newborn children in Suzhou, regardless of residence, are immunized; all immunizations are logged in the program database.

### Estimating Incidence of Hospitalization for IPD

**Figure 3:**
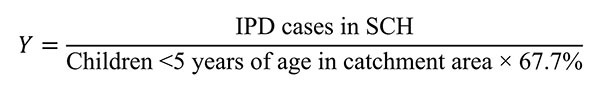
Equation for the incidence of hospitalization for IPD in the catchment area of SCH.

We used a rapid method from the World Health Organization to calculate the incidence of hospitalization for IPD (*11*). This method uses the sentinel hospital’s meningitis surveillance system to identify the numerator as potential bacterial meningitis cases and the denominator as the estimated number of children at risk for meningitis in the catchment area. China does not have a nationwide meningitis surveillance system, but SCH collects routine CSF, blood, and pleural fluid cultures from children with suspected IPD. We estimated the denominator (i.e., children at risk for IPD in the area) by multiplying the population <5 years of age in the catchment area by the percentage of children in that age group who are treated at SCH (67.7%). Thus, we calculated the incidence of hospitalization for IPD in the catchment area of SCH (*Y*) as (Figure 3)

We used the Wilson method for binominal distribution to estimate 95% CIs of IPD hospitalizations. We used the χ^2^ test to compare the incidence of hospitalization across different age groups. We conducted all statistical analyses using SAS version 9.4 (SAS Institute, https://www.sas.com).

## Results

### Characteristics of IPD Patients

During January 2010–December 2017, SCH collected 20,260 CSF specimens from children <5 years of age. Among the specimens, 283 tested positive for bacterial infections, including 46 (16.3%) positive for *S. pneumoniae* and 51 (18.0%) positive for *Staphylococcus epidermidis*. SCH also collected 62,858 blood and 138 pleural effusion specimens for bacterial detection from children <5 years of age. Of these samples, 2,432 blood and 29 pleural effusion cultures tested positive for bacterial infection. *S. pneumoniae* was the sixth most common bacteria in the positive blood cultures (110/2,432; 4.5%) and the most common bacteria in the positive pleural effusion cultures (16/29; 55.2%). The proportion of samples that tested positive for *S. pneumoniae* from children <5 years of age were 0.2% (46/20,260) in CSF, 0.2% (110/ 62,858) in blood, and 11.6% (16/138) in pleural effusion samples. After accounting for duplicate specimens, SCH identified 105 patients <5 years of age with IPD during 2010–2017. The male:female ratio was 1.3; 54.3% were 2–<5 years of age. Common diagnoses included meningitis (31.4%), pneumonia (28.6%), and sepsis (21.0%) (Table 1).

**Table 1:** Characteristics of children with invasive pneumococcal diseases, Suzhou, China, 2010–2017

Characteristic	Age group, no. (%)			
	<1 y	1–<2 y	2–<5 y	Total
Total	31 (100.0)	17 (100.0)	57 (100.0)	105 (100.0)
Sex				
M	17 (54.8)	10 (58.8)	33 (57.9)	60 (57.1)
F	14 (45.2)	7 (41.2)	24 (42.1)	45 (42.9)
Year				
2010	5 (16.1)	1 (5.9)	4 (7.0)	10 (9.5)
2011	4 (12.9)	4 (23.5)	3 (5.3)	11 (10.5)
2012	4 (12.9)	1 (5.9)	5 (8.8)	10 (9.5)
2013	5 (16.1)	4 (23.5)	4 (7.0)	13 (12.4)
2014	4 (12.9)	4 (23.5)	9 (15.8)	17 (16.2)
2015	5 (16.1)	1 (5.9)	12 (21.1)	18 (17.1)
2016	2 (6.5)	1 (5.9)	6 (10.5)	9 (8.6)
2017	2 (6.5)	1 (5.9)	14 (24.6)	17 (16.2)
Primary discharge diagnosis				
Meningitis	16 (51.6)	4 (23.5)	13 (22.8)	33 (31.4)
Pneumonia	7 (22.6)	3 (17.6)	20 (35.1)	30 (28.6)
Sepsis	6 (19.4)	6 (35.3)	10 (17.5)	22 (21.0)
Upper respiratory infection	1 (3.2)	1 (5.9)	1 (1.8)	3 (2.9)
Bronchitis	0	1 (5.9)	1 (1.8)	2 (1.9)
Other*	1 (3.2)	2 (11.8)	12 (21.1)	15 (14.3)

*Other conditions: leukemia (6 cases), anemia (1 case), endocarditis (2 cases), arrhythmia (2 cases), acute otitis media (1 case), jaundice (1 case), acute gastritis (1 case), and psoas abscess (1 case).

### Incidence of Hospitalization for Children with IPD

**Figure 1:**
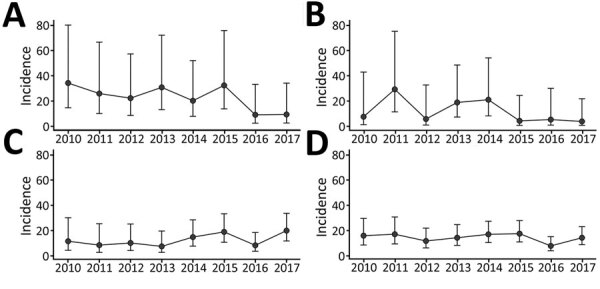
Estimated invasive pneumococcal diseases hospitalization incidence per 100,000 children among children <5 years of age, by age and year, Suzhou, China, 2010–2017. A) Children <1 year of age; B) children 1–<2 years of age; C) children 2–<5 years of age; D) children <5 years of age. Error bars indicate 95% CIs.

The population of children <5 years of age in the catchment area increased from 90,756 in 2010 to 171,676 in 2017. During January 2010–December 2017, the estimated annual incidence of hospitalization for IPD among children in this age group in Suzhou ranged from 8.16 to 17.86 per 100,000 children, peaking in 2015 (Table 2). The incidence of hospitalization for IPD fluctuated without significance (χ^2^ = 1.51; p>0.05) (Figure 1). The IPD hospitalization incidence differed among age groups and was highest among children <1 year of age (χ^2^ = 6.73; p<0.05). The incidence of hospitalization among children <5 years of age was 4.57 (95% CI 3.26–6.42)/100,000 children for meningitis, 4.16 (95% CI 2.91–5.93)/100,000 children for bacteremic pneumonia, and 3.05 (95% CI 2.01–4.62)/100,000 children for sepsis.

**Table 2:** Estimated invasive pneumococcal diseases hospitalization incidence among children <5 years of age, Suzhou, China, 2010–2017*

Year	Children <1 y of age				Children 1–<2 y of age				Children 2–<5 y of age		
	IPD cases at SCH	Pop.†	HI‡ (95% CI)		IPD cases at SCH	Pop.†	HI‡ (95% CI)		IPD cases at SCH	Pop.†	HI‡ (95% CI)
2010	5	21,510	34.33 (14.67–80.35)		1	19,433	7.60 (1.34–43.02)		4	49,813	11.86 (4.61–30.51)
2011	4	22,731	25.99 (10.11–66.81)		4	20,136	29.34 (11.41–75.43)		3	50,527	8.77 (2.98–25.79)
2012	4	26,452	22.35 (8.69–57.46)		1	25,483	5.80 (1.02–32.83)		5	70,423	10.49 (4.48–25.54)
2013	5	23,893	30.91 (13.20–72.35)		4	31,228	18.92 (7.36–48.65)		4	76,041	7.77 (3.02–19.98)
2014	4	29,110	20.30 (7.89–52.18)		4	28,006	21.10 (8.21–54.26)		9	87,752	15.15 (7.97–28.79)
2015	5	22,745	32.48 (13.87–76.02)		1	34,119	4.33 (0.76–24.53)		12	92,038	19.26 (11.02–33.66)
2016	2	32,360	9.13 (2.5–33.28)		1	27,768	5.32 (0.94–30.13)		6	102,818	8.62 (3.95–18.80)
2017	2	31,415	9.40 (2.58–34.28)		1	38,255	3.86 (0.68–21.88)		14	102,006	20.27 (12.07–34.02)
Total	31	210,216	21.78 (15.34–30.91)		17	224,428	11.19 (6.99–17.92)		57	631,418	13.33 (10.29–17.27)

*Incidence is cases/100,000 children. HI, hospitalization incidence; IPD, invasive pneumococcal disease; pop., population; SCH, Suzhou University Affiliated Children Hospital. †Population <5 years of age in Suzhou (5 municipal districts).  ‡Hi calculated by dividing no. IPD cases at SCH by 67.7% of population <5 years of age in Suzhou.

### Serotype Distribution and Incidence of IPD Hospitalizations by Serotype

SCH sent 74 (70.5%) pneumococcal isolates from patients with IPD to the laboratory for serotyping. We identified 8 different capsular serotypes: 6B (21/74; 28.4%), 14 (14/74; 18.9%), 19A (14/74; 18.9%), 19F (9/74; 12.2%), 23F (8/74; 10.8%), 20 (3/74; 4.1%), 9V (3/74; 4.1%), and 15B/C (2/74; 2.7%). Thus, the serotype coverage rate, or the percentage of cases caused by serotypes preventable by vaccination, of PCV10 was 74.4% and PCV13 was 93.2%.

**Figure 2:**
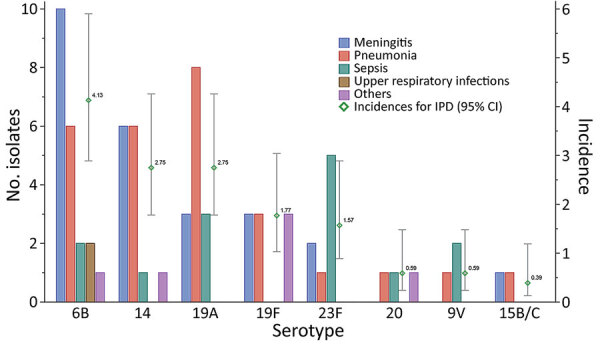
Association of serotype of *Streptococcus pneumoniae* with primary discharge diagnosis (data bars) and serotype-specific hospitalization incidence (data points, cases per 100,000 children) of children <5 years of age, Suzhou, China, 2010–2017. Error bars indicate 95% CIs.

Among patients with meningitis, serotypes 6B (10/25; 40.0%) and 14 (6/25; 24.0%) were most common. Of patients with sepsis, the most common serotypes were 23F (5/14; 35.7%) and 19A (3/14; 21.4%) (Figure 2). However, these findings were not significant. We calculated the estimated serotype-specific IPD hospitalization incidences to be 4.13 (95% CI 2.89–5.90)/100,000 children for 6B, 2.75 (95% CI 1.78–4.26)/100,000 children for 14, 2.75 (95% CI 1.78–4.26)/100,000 children for 19A, 1.77 (95% CI 1.03–3.04)/100,000 children for 19F, 1.57 (95% CI 0.89–2.89)/100,000 children for 23F, 0.59 (95% CI 0.24–1.48)/100,000 children for 20 and 9V, and 0.39 (95% CI 0.13–1.19)/100,000 children for 15B/C (Figure 2).

## Discussion

We describe children hospitalized for IPD at SCH during January 2010–December 2017. We used previous studies (*12*–*14*) to estimate the incidence of hospitalization for IPD among children <5 years of age in Suzhou. During January 2010–December 2017, the annual incidence of hospitalization for IPD in Suzhou ranged from 8.16 to 17.86/100,000 children and the average incidence was 14.55/100,000 children. The annual incidence of IPD did not significantly change during the 8-year period, peaking at 17.86/100,000 children in 2015. The IPD hospitalization incidence was highest (21.78 hospitalizations/100,000 children) among those <1 year of age and lowest (13.33 hospitalizations/100,000 children) among those 2–<5 years of age.

We estimated the incidence of hospitalization for pneumococcal meningitis as 4.57/100,000 children. A systematic review in China estimated the mean incidence of pneumococcal meningitis in children 1–23 months of age as 5.1 cases/100,000 children during 1999–2003 (*16*). Another study in China estimated the incidence of bacterial meningitis as 6.95–22.30 cases/100,000 children <5 years of age during 2006–2009; *S. pneumoniae* caused 53% of these cases (*10*), indicating a pneumococcal meningitis incidence of ≈3.7–11.8/100,000 children. This incidence is comparable to our estimates. A study in Beijing estimated the incidence of sepsis in adults as 461 cases/100,000 persons (*17*), but that study did not classify patients on the basis of bacterial infection. Many studies around the world indicate similar incidences of IPD before the use of PCVs (*18*–*20*). A 20-year study in Atlanta, Georgia, USA, showed that during 2003–2013, the overall IPD rate was 8.03–14.02 cases/100,000 persons; incidence was also estimated by using *S. pneumoniae* isolates collected from normally sterile sites (*4*). The IPD incidence of children <5 years of age in India in 2006 was 1,500 cases/100,000 persons (*5*), much higher than in China and other countries.

The proportion of CSF, blood, and pleural effusion samples that tested positive for *S. pneumoniae* were lower than those found in studies in India and Turkey (*21*,*22*). Possible reasons for the lower proportion of *S. pneumoniae*–positive samples included the common use of antimicrobial drugs before hospital admission (*7*,*10*) and the use of only traditional bacterial cultures, which are less sensitive than immunochromatography and PCR, for identification of *S. pneumoniae*. We did not collect data on antimicrobial drug usage, but our previous study in the same hospital during 2006–2007 found that ≈86% of children with lower respiratory tract infections used antimicrobial drugs, not all of which were prescribed, before admission (*23*). Li et al. showed that, in China’s general hospitals, the percentage of outpatients treated with prescribed antimicrobial drugs declined from 20.17% in 2012 to 12.94% in 2016 (*24*). Since 2012, China has improved the judicious use of antimicrobial drugs; however, the inappropriate use of antimicrobial drugs in children remains a serious problem (*25*,*26*). We might underestimate the incidence of hospitalization for IPD because pretreating with antimicrobial drugs at home might reduce the proportion of IPD patients who test positive for *S. pneumoniae*.

In this study, we found that serotypes 6B, 14, and 19A caused 66.2% of all IPD infections. These results differ from those of our recent systematic review (*27*), which found 19F and 19A to be the most common serotypes among invasive strains isolated from children in mainland China during 2000–2016. Furthermore, a study in England and Wales found serotype 18C to be the most common serotype among children <5 years of age with meningitis (*28*). Therefore, we believe the distribution of pneumococcal serotypes varies with geographic location.

PCVs have been introduced in >100 countries, possibly contributing to a major reduction in the incidence of IPD (*29*). During most of the study period, PCV7 was the only PCV available in mainland China; whether this vaccination decreased the incidence of IPD in China is unknown. PCV7 uptake in China was very low, an estimated 2%–7% (*8*,*9*). Our previous study showed that 87.4% of IPD cases among children in mainland China (*27*) were caused by pneumococcal serotypes preventable by PCV13; in the study we report here, that proportion increased to 93.2%. Therefore, if the vaccine uptake of PCV13 in China is sufficiently high, we expect similar reductions in the incidence of IPD in children in China, as has been observed in other countries (*29*).

The major limitation of this study is that common antimicrobial drug use might have reduced the proportion of specimens that tested positive for *S. pneumoniae*, causing us to underestimate the IPD hospitalization incidence. In addition, SCH only took blood cultures of patients with a temperature >39.0°C; this policy might overlook IPD patients without fever. Furthermore, we did not have data on IPD patients in Suzhou who were not treated at SCH. Because SCH treated 67.7% of children <5 years of age who were discharged from hospitals in downtown Suzhou in 2011 (*13*), we used this proportion to estimate the number of children at risk for IPD in Suzhou; we might have overestimated or underestimated the incidence of IPD. Our previous study found that ≈50% of children with influenza-like illness in the catchment area are treated at SCH (*12*). Because SCH is the only pediatric tertiary hospital in Suzhou and IPDs are more severe than influenza-like illnesses, we assumed that the proportion of children with IPD in the catchment area who were treated at SCH would be >50%. Finally, the small number of identified IPD cases might skew our estimates.

In conclusion, we found that the incidence of hospitalization for IPD in Suzhou was similar to that of populations in China and other countries before the use of PCVs. In addition, the IPD hospitalization incidence did not significantly change during 2010–2017. In China, a high proportion of pneumococcal serotypes are preventable with PCV13. These results provide baseline data of IPD incidence before PCV13 use in China.
